# Development of Neuropeptide Y and Cell-Penetrating Peptide MAP Adsorbed onto Lipid Nanoparticle Surface

**DOI:** 10.3390/molecules27092734

**Published:** 2022-04-24

**Authors:** Sara Silva, Joana Marto, Lídia M. Gonçalves, Henrique S. Fernandes, Sérgio F. Sousa, António J. Almeida, Nuno Vale

**Affiliations:** 1 OncoPharma Research Group, Center for Health Technology and Services Research (CINTESIS), Rua Dr. Plácido da Costa, 4200-450 Porto, Portugal; saracpsilva21@gmail.com; 2 Faculty of Pharmacy, University of Porto, Rua de Jorge Viterbo Ferreira 228, 4050-313 Porto, Portugal; 3 Research Institute for Medicines (iMed.ULisboa), Faculty of Pharmacy, University of Lisbon, Av. Prof. Gama Pinto, 1649-003 Lisbon, Portugal; jmmarto@ff.ulisboa.pt (J.M.); lgoncalves@ff.ulisboa.pt (L.M.G.); aalmeida@ff.ulisboa.pt (A.J.A.); 4 UCIBIO/REQUIMTE, BioSIM, Departamento de Medicina, Faculdade de Medicina da Universidade Do Porto, Alameda Prof. Hernâni Monteiro, 4200-319 Porto, Portugal; hfernandes@med.up.pt (H.S.F.); sergiofsousa@med.up.pt (S.F.S.); 5 Associate Laboratory i4HB, Institute for Health and Bioeconomy, Faculdade de Medicina, Universidade do Porto, 4200-319 Porto, Portugal; 6 Department of Community Medicine, Information and Health Decision Sciences (MEDCIDS), Faculty of Medicine, University of Porto, Rua Dr. Plácido da Costa, s/n, 4200-450 Porto, Portugal; 7 Associate Laboratory RISE, Health Research Network, Faculty of Medicine, University of Porto, Al. Prof. Hernâni Monteiro, 4200-319 Porto, Portugal

**Keywords:** cell-penetrating peptide, neuropeptide Y, nanoparticles, adsorption, potential zeta, Tacrine

## Abstract

Functionalization of nanoparticles surfaces have been widely used to improve diagnostic and therapeutic biological outcome. Several methods can be applied to modify nanoparticle surface; however, in this article we focus toward a simple and less time-consuming method. We applied an adsorption method on already formulated nanostructured lipid carriers (NLC) to functionalize these nanoparticles with three distinct peptides sequences. We selected a cell-penetrating peptide (CPP), a lysine modified model amphipathic peptide (Lys(N_3_)-MAP), CPP/drug complex, and the neuropeptide Y. The aim of this work is to evaluate the effect of several parameters such as peptide concentration, different types of NLC, different types of peptides, and incubation medium on the physicochemical proprieties of NLC and determine if adsorption occurs. The preliminary results from zeta potential analysis indicate some evidence that this method was successful in adsorbing three types of peptides onto NLC. Several non-covalent interactions appear to be involved in peptide adsorption with the possibility of three adsorption peptide hypothesis that may occur with NLC in solution. Moreover, and for the first time, in silico docking analysis demonstrated strong interaction between CPP MAP and NPY Y1 receptor with high score values when compared to standard antagonist and NPY.

## 1. Introduction

Nanotechnology has been widely investigated in many science fields. In part due to the major advantages that arise from its use. In terms of drug delivery, nanoparticles (NPs) can enhance drug solubility, increase bioavailability, enhance permeation across biological membranes, enable steady drug release, promote targeting and protection against metabolization and degradation [1,2]. Lipid NPs have been showing great potential as drug delivery systems allied with low toxicity and use of biodegradable components in the formulation such as triglycerides and phospholipids [3].

Neuropeptide Y (NPY) is a 36-amino-acid neuropeptide that is synthetized, processed, and stored in neurons [4]. NPY is highly expressed on central and peripheral neurons but is more prevalent in cortical areas, limbic system, amygdala, hippocampus, hypothalamus, and more [5,6]. In addition, NPY can act as a neurotransmitter or neuromodulator and displays multiple functions including balance of food intake [7], energy metabolism, blood pressure, regulate smooth muscle tone, balance with cognitive functions, and more [8,9,10]. Different peptide fragments can arise from NPY mRNA sequence and its expression of NPY can be affected by classic neurotransmitters, steroid hormones, and growth factors [10,11] and is mainly found in GABA neurons. For instance, C-terminal amphipathic end as the most structural importance, α-helix is important for receptor binding selectivity and its biological effect and polyproline type II helix is linked with secondary and tertiary structure stability [8]. At molecular level, NPY exerts its functions through binding and activation of different receptors such as Y1, Y2, Y4, Y5, and Y6 that are coupled with G-proteins [9]. Although NPY has been described with the main functions in recent research papers, it has become clearer that NPY can exert a wide range of molecular functions crucial to neuronal protection and proliferation [6]. Several studies have demonstrated a close relationship of NPY with inhibition of noradrenaline release and inhibition of voltage-dependent Ca^2+^ channel resulting in a decrease in Ca^2+^ intracellular influx [12]. Moreover, NPY can modulate neurogenesis and neurotrophins [13,14], decrease excitotoxicity [15], regulate calcium homeostasis [12], and attenuate neuroinflammation [16], making a good candidate as a therapeutic molecule against central nervous systems disorders (anxiety, epilepsy, depression, ischemia), and neurodegenerative diseases.

The increase of world population life-spam neurodegenerative diseases is becoming more prevalent. Several studies have shown that NPY is a key neurological biomarker demonstrating NPY mRNA expression, and NPY binding sites are affected in different ways for each neurological disease. For instance, in Alzheimer disease (AD), researchers observed decreased NPY levels and NPY receptors in patients’ brain (cerebral cortex and hippocampus), decreased NPY levels in cerebrospinal fluid (CSF), and decreased NPY levels in peripheral plasma. This decrease is associated with the neurodegeneration progress. In Parkinson’s disease (PD) patients, the opposite was observed; that is an increase in NPY mRNA expression levels on striatum and this phenom its related with an endogenous protective response mechanism to neurodegeneration occurring [17]. In a study conducted by Croce and his team, a close link between neurotrophins expression and NPY treatment was demonstrated. The authors exposed both neuroblastoma cell line and primary neuron cells to toxic concentrations of amyloid beta peptide and NPY treatment leading to neuroprotection, increased neurotrofins expression, and promoted cell survival [13,14]. Another study demonstrated that NPY delivery to the brain of AD model mice resulted in a decrease of neurodegeneration and improvement in memory [18]. Furthermore, Decressac and his team demonstrated that NPY injection led to neuroprotection of nigral dopamine neurons through Y2 activation in PD mice models [19]. The authors also modified the disease course of Huntington’s disease transgenic mice [20].

Functionalization of NPs surface can be a useful technique to enhance the potential of the nanosystem in development. These surface modifications include conjugation of different molecules of interest such as chemicals, active biomolecules, oligonucleotides, peptides, antibodies, aptamers, and more [21]. This strategy enables linkage of diagnostic, therapeutic, and theragnostic molecules that promote targeting, cellular delivery, and avoid degradation [22]. Conjugation of molecules on NPs surface can occur through physical absorption with non-covalent interactions or by direct binding through covalent conjugation. Focusing on physical adsorption, the molecule of interest is linked to the surface by weak interactions which includes electrostatic interactions, hydrogen binding, hydrophobic, and Van der Waals attractive forces [23]. This method is simple, less time-consuming, and do not require NPs or molecules modifications [23].

For instance, there are some peptides that can be used to improve transport across membranes through energy-independent pathways [24], also known as cell-penetrating peptides (CPP). CPP are characterized as short peptides with less than 30 amino acid residues with the ability to interact with cellular membrane promoting endocytosis. Furthermore, CPP can form CPP/cargo complexes and successfully deliver to the cell’s different types of drugs, biomolecules, NPs, and liposomes against a variety of diseases [25]. MAP, a model amphipathic peptide, was developed with special design to ensure amphipathic structure and high membrane cellular interaction. [26,27]. The CPP MAP, under the right conditions, demonstrates high cellular penetration through multiple mechanisms (non-endocytic and endocytic) [27,28] and delivers different cargo into cell compartments [29,30,31,32].

Molecular docking is becoming a crucial tool in drug discovery to find and optimize lead compounds and can also be applied to predict in silico experimental binding mode and affinity of a selective molecule to a binding site of the receptor of interest [33]. During computational docking experiment, the computer generates several poses and compares scoring to the target molecule. The poses can be accepted or rejected based on the score obtain [34]. Moreover, different programs can be employed for high throughput docking of a large database of molecules such as AutoDock Vina, DOCK, FlexX, GOLD, ICM [34].

**Scheme 1:**
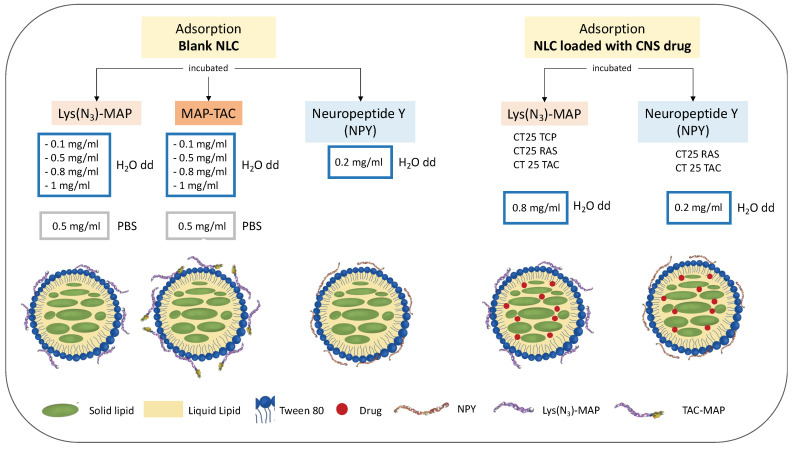
Graphical representation of adsorption method applied with the three peptides, NLCs, different concentrations, and medium conditions conducted in this project.

In the first part of this article, we apply an adsorption method to functionalize three different peptides onto nanostructured lipid carrier (NLC) surface and conduct an initial NP characterization to determine some evidence of peptide absorption (Scheme 1). We demonstrated how adsorption of NPY, an CPP lysine-modified MAP [Lys(N_3_)-MAP] and CPP/cargo complex (Tacrine-MAP) can affect particle size, polydispersity index (PDI), and zeta potential. In the second part of this study, we conducted an in silico receptor molecular docking assay to evaluate possible MAP interaction with NPY Y1 receptor and better understand a possible future interaction in cells with these membrane receptor present.

## 2. Results

### 2.1. CPP and CPP/Drug Complex Adsorption onto NLC Surface

#### 2.1.1. Influence of Different Lys(N_3_)-MAP Concentrations

**Figure 1:**
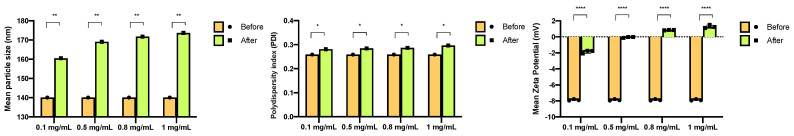
Particle size, polydispersity index, and zeta potential of blank CT25 NLC before and after adsorption experiments with different peptide concentrations of Lys(N_3_)-MAP at 37 °C with incubation in ultra-purified double distilled water (H_2_O dd); mean ± SD, *n* = 3. Statistical analysis between the control groups was performed using two-way ANOVA with Tukey’s multiple comparisons test (* *p* < 0.05; ** *p* < 0.01; **** *p* < 0.0001).

After Lys(N_3_)-MAP incubation with increased concentrations, we can observe some gradual increased changes to the particle size and zeta potential (Figure 1 and Table 1). The data show after Lys(N_3_)-MAP incubation the CT25 Blank (CT25 B) NLC formulation significantly increased the size from 140 nm to up to 170 nm at a higher concentration of 1 mg/mL. The graphic shows a sustained increase of CT25 B size depending on the amount of Lys(N_3_)-MAP use. On the other hand, PDI results in a slight increase after peptide incubation. For instance, the zeta potential values demonstrated significant differences dependent on concentration. CT25 B incubation with 0.1 mg/mL, 0.5 mg/mL, 0.8 mg/mL, and 1 mg/mL resulted in a zeta potential change from −7.88 mV to −1.89 mV, −0.05 mV, 0.85 mV, and 1.27 mV, respectively.

#### 2.1.2. Influence of Lys(N_3_)-MAP Adsorbed into Drug Loaded NLC

**Figure 2:**
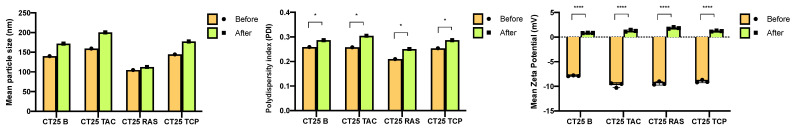
Particle size, polydispersity index and zeta potential of CT25 B and drug load CT25 NLC before and after adsorption experiments with 0.8 mg/mL Lys(N_3_)-MAP at 37 °C with incubation in H_2_O dd; mean ± SD, *n* = 3. Statistical analysis between the control groups was performed using two-way ANOVA with Tukey’s multiple comparisons test (* *p* < 0.05; **** *p* < 0.0001).

When analyzing the influence of different drug-loaded NLC formulations with the fixed concentration of 0.8 mg/mL of Lys(N_3_)-MAP, the results showed similar changes before and after incubation (Figure 2 and Table 1). There was no significant increase in the NLC particle size, and a significant increase in PDI values was found. For CT25 loaded with Tacrine (CT25TAC) before incubation particle size and PDI were 159.5 nm and 0.258, respectively and after Lys(N_3_)-MAP incubation the values were 200.5 nm and 0.305. The CT25 loaded with RAS (CT25RAS) before incubation particle size and PDI were 104.8 nm and 0.210 and after Lys(N_3_)-MAP incubation the values were 112.6 nm and 0.251, respectively. Tranylcypromine-loaded CT25 (CT25TCP) before incubation particle size and PDI were 146.5 nm and 0.254 and after Lys(N_3_)-MAP incubation the values were 158.3 nm and 0.288, respectively. As expected, the zeta potential after incubation switched from negative to positive in all CT25 drug-loaded NLC formulations.

#### 2.1.3. Influence of Different TAC-MAP Concentrations

**Figure 3:**
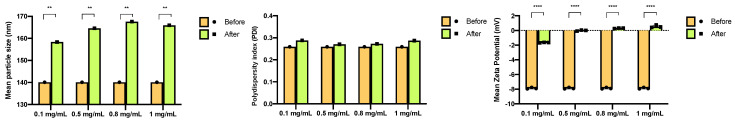
Particle size, polydispersity index and zeta potential of blank CT25 NLC before and after adsorption experiments with different TAC-MAP concentrations at 37 °C with incubation in H_2_O dd; mean ± SD, *n* = 3. Statistical analysis between the control groups was performed using two-way ANOVA with Tukey’s multiple comparisons test (** *p* < 0.01; **** *p* < 0.0001).

This analysis was conducted to evaluate the influence of crescent concentration of TAC-MAP on CT25 B physicochemical parameters. The data showed a significant increase in CT25 B particle size after incubation, values ranging from 140 nm before incubation to up to 165.9 nm after TAC-MAP incubation (Figure 3). PDI values indicate no significant change in CT25 B formulation after TAC-MAP incubation independent of the concentration used. The mean zeta potential analysis demonstrated significant increase in CT25 B surface charge after TAC-MAP incubation dependent on concentration applied. Control CT25 B showed a zeta potential of −7.88 mV, whereas 0.1 mg/mL resulted in an increase to −1.65 mV; 0.5 mg/mL to 0.16 mV; 0.8 mg/mL to 0.30 mV; and 1 mg/mL to 0.55 mV.

#### 2.1.4. Influence of CT25 B Amount on TAC-MAP Adsorption

**Figure 4:**
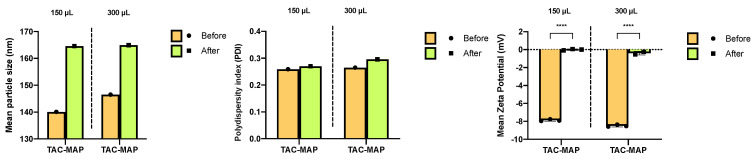
Particle size, polydispersity index and zeta potential of different initial amount of blank CT25 NLC before and after adsorption experiments with 0.5 mg/mL TAC-MAP at 37 °C with incubation in H_2_O dd; mean ± SD, *n* = 3. Statistical analysis between the control groups was performed using two-way ANOVA with Tukey’s multiple comparisons test (**** *p* < 0.0001).

Another parameter was assessed to determine the influence of the quantity of CT25 B NLC in solution when the same concentration of TAC-MAP was applied. The data showed that the increase in the amount of CT25 B in solution lead to the same differences in characterization values before and after TAC-MAP incubation (Figure 4).

#### 2.1.5. Influence of Incubation Medium H_2_O vs. PBS on Peptide Adsorption

**Figure 5:**
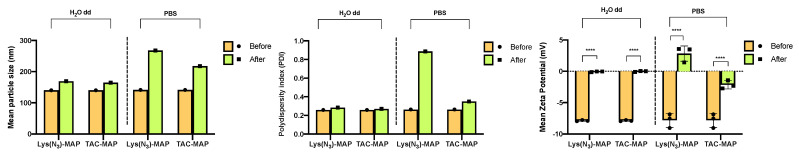
Particle size, polydispersity index, and zeta potential of blank CT25 NLC before and after adsorption experiments with 0.5 mg/mL Lys(N_3_)-MAP and 0.5 mg/mL TAC-MAP at 37 °C with different incubation medium H_2_O dd and PBS; mean ± SD, *n* = 3. Statistical analysis between the control groups was performed using two-way ANOVA with Tukey’s multiple comparisons test (**** *p* < 0.0001).

In order to predict a more physiological context, we compared the differences obtain from incubation of CT25 B with 0.5 mg/mL of Lys(N_3_)-MAP and TAC-MAP in phosphate buffered solution (PBS pH 7.4). From this analysis, the results demonstrated some differences between the two incubation mediums (Figure 5). For instance, particle size increases substantially in both peptides when incubated with PBS medium. After Lys(N_3_)-MAP was incubated with CT25 B in H_2_O dd medium, the particle size increased around 20%, on the other hand, PBS medium incubation resulted in 90% particle size increase. For the PDI and zeta potential the increase was higher, after Lys(N_3_)-MAP incubation on PBS the values increased around 236% and 10.65 mV, respectively (Figure 5). After TAC-MAP was incubated with CT25 B in H_2_O dd medium the particle size increased around 18%, on the other hand, PBS medium incubation resulted in 54% particle size increase. For the PDI and zeta potential the same increase was observed, after TAC-MAP incubation on PBS the values increased around 33% and 5.66 mV, respectively (Figure 5).

### 2.2. Neuropeptide Y Adsorption onto NLC Surface

**Figure 6:**
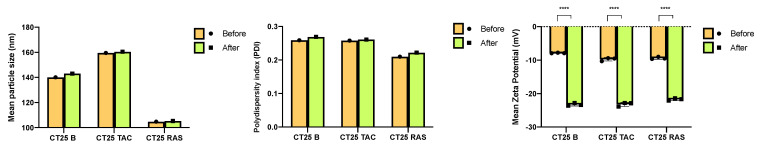
Particle size, polydispersity index, and zeta potential of CT25 B and drug load CT25 NLC before and after adsorption experiments with NPY at 37 °C with incubation in H_2_O dd; mean ± SD, *n* = 3. Statistical analysis between the control groups was performed using two-way ANOVA with Tukey’s multiple comparisons test (**** *p* < 0.0001).

Potential therapeutic peptide NPY was incubated with CT25 B and drug-loaded CT25 NLC. The characterization on its particle size, PDI, and zeta potential is expressed in Figure 6 and Table 1. The results showed similar particle size and PDI values before and after NPY incubation. Although, zeta potential analysis after NPY incubation resulted in a decrease in all NLC tested, independent of the type of drug encapsulated. For the CT25 B there was a decrease in zeta potential from −7.88 mV to −23.27 mV; for CT25TAC the zeta potential decreased from −9.73 mV to −25.2 mV; and for CT25RAS from −9.39 mV to −21.73 mV.

### 2.3. Receptor Molecular Docking Assays

**Figure 7:**
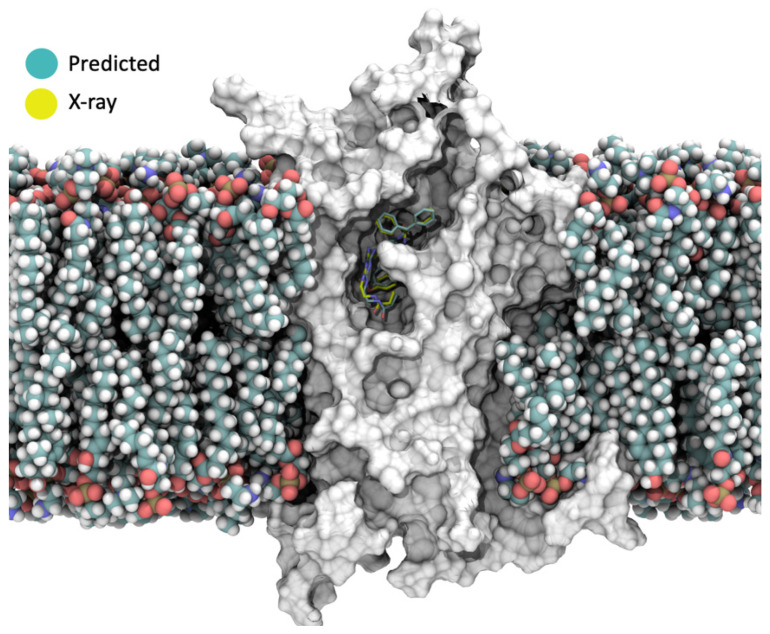
Validation of the docking protocol by redocking UR-MK299 against NPY1R. In cyan, the conformation predicted from docking. In yellow, the X-ray crystallography conformation. Slab-view representation, showing the plane of the interior binding pocket.

Figure 7 illustrates the comparison of the pose predicted using the optimized docking protocol in redocking UR-MK299 antagonist against the experimental NPY1R/UR-MK299 complex (PDB structure 5ZBQ). As can be seen, the optimized docking protocol is able to accurately reproduce the experimental structure, with a root-mean-square deviation (RMSD) of 0.86 Å, demonstrating its robustness.

Table 2 presents the docking scores obtained with GOLD/PLP in docking MAP and NPY against NPY1R. The value obtained for the reference ligand, the UR-MK299 antagonist, is presented for comparison. The GOLD/PLP scoring function is dimensionless and scores higher values to molecules with the highest affinity [35]. Values above 60 are normally considered to be indicative of strong association [36].

The values presented in Table 2 for MAP and NPY suggest that both peptides are able to strongly interact with the NPY1R receptor, with scores of 101.29 and 95.12, respectively. These values are very high and close to the docking score obtained for the reference antagonist ligand UR-MK299, reinforcing their potential as NPY1R binders.

**Figure 8:**
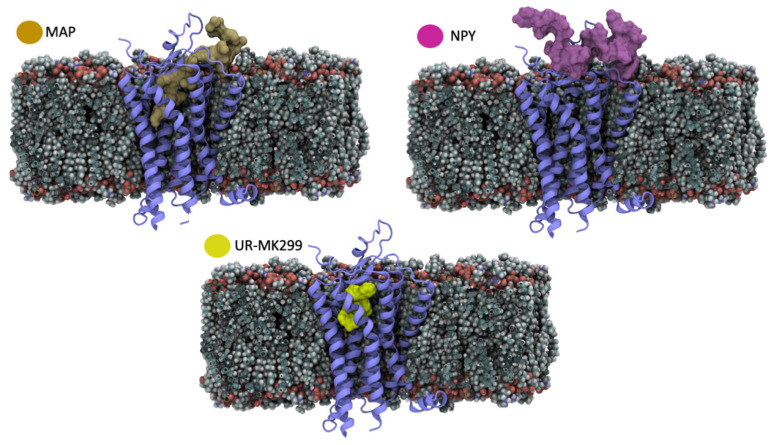
Representation of the volume occupied by MAP, NPY, and antagonist UR-MK299 when complexed with NPY1R, illustrating the different binding modes predicted for the two peptides and the one observed in the X-ray of the complex formed with the antagonist molecule.

The results suggest that MAP is able to bind with higher affinity to NPYR1 than NPY. Figure 8 shows the predicted dominant binding modes of MAP and NPY. As can be seen in Figure 8, MAP is able to insert tightly inside NPYR1, occupying the binding pocket that was initially occupied by UR-MK299, and filling the most important interactions established by this antagonist.

For NPY a similar binding mode was not identified. Nevertheless, the results show that NPY has the ability to establish strong interactions with the outer loops of NPY1R.

**Figure 9:**
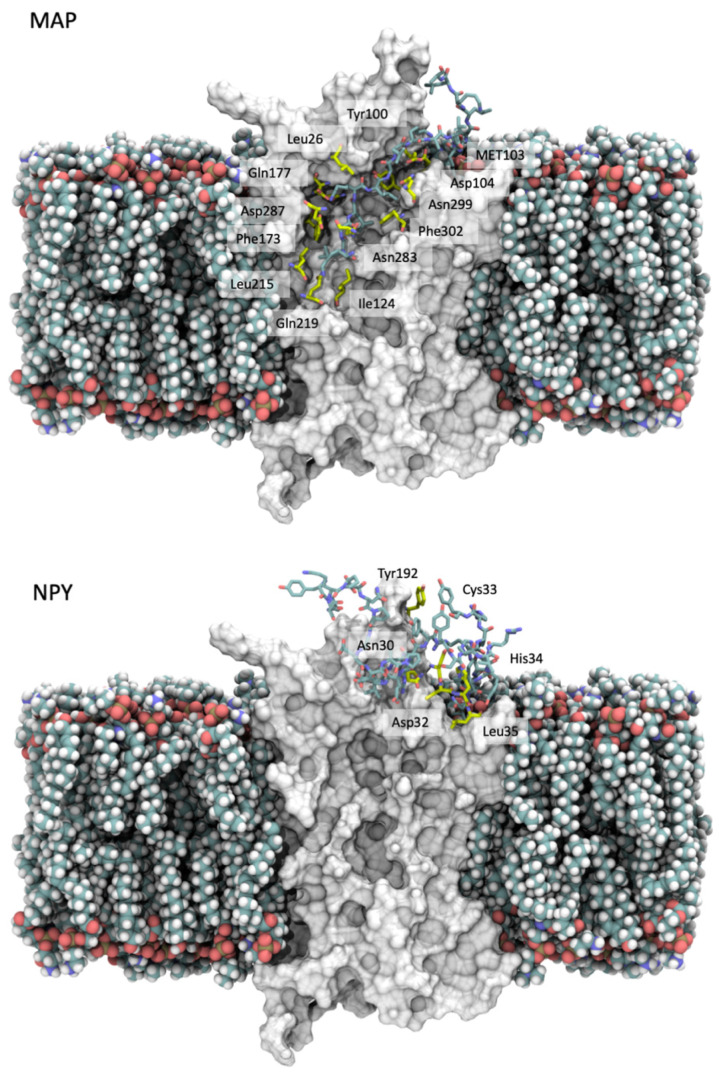
Slab-view representation of the interaction of MAP and NPY with NPY1R, illustrating the most important interactions established.

Figure 9 represents the main interactions established between MAP and NPY1R. MAP interacts preferentially through its N-terminal amino acid residues, with its Lys1-Lys9 portion inserting deep into the NPYR1 inside cavity. It forms important interactions with Gln219, Asn283 and Asp287, and Gln177. In addition, it establishes hydrophobic contacts with Leu26, Ile124, Phe173, and Phe302. Other relevant interactions were observed with Tyr100, Met103, Asp104, Leu215, and Asn299, near the cavity exit.

## 3. Discussion

Molecular interactions are important to understand and determined certain biological phenomena such as peptide adsorption, cell adhesion, and biological membrane interaction. Adsorption methods have been widely applied to functionalize different NPs with a variety of molecules. Some of them have been coated with PEG, Tween 80, antibodies, peptides, and even drugs for different purposes such as disease diagnostic, treatment, and delivery [37,38,39,40,41]. In this project, we conducted three peptide adsorption experiments onto NLC surface and observed the physicochemical changes occurring on NLC suspension in terms of particle size, PDI, and zeta potential through dynamic light scattering analyses and anemometry. Zeta potential analysis is crucial for the characterization of NP-based drug delivery systems. From the data, we can deduce the stability of NPs in suspension. A high value of zeta potential ±30 mV is linked with repulsive electrostatic forces between NPs which is related with higher stability and decreased NP aggregation in suspension [42]. The zeta potential can also vary depending on pH of the suspension medium. The influence of pH can be determined by calculation of the isoelectric point. Moreover, zeta potential can be a powerful indicator for the characterization of NPs surface overall charge and indicates a possible molecule adsorption [43].

**Scheme 2:**
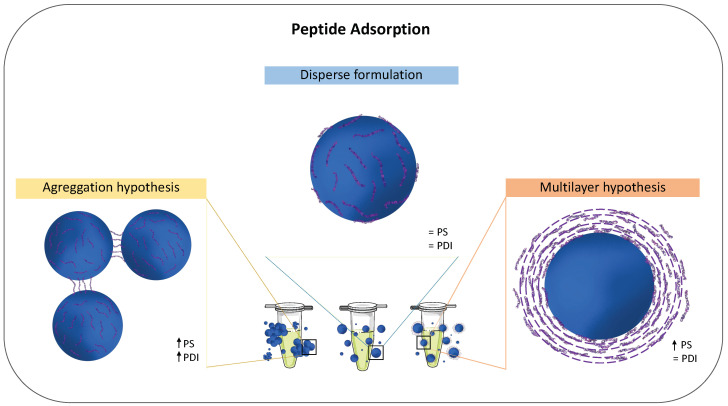
Schematic representation of three hypothesis occurring through the data obtain in this project to explain the differences on particle size and PDI observed.

In this article several parameters were evaluated to ensure a protocol optimization and determine the differences that can occur in different method conditions. The data obtain from increased peptide concentrations in NLC incubation demonstrated a concentration-dependent increase in particle size, PDI, and zeta potential for both Lys(N_3_)-MAP and TAC-MAP. The increase of particle size could indicate the formation of a multilayer peptide on CT25 B surface (Scheme 2—multilayer hypothesis), already observed in other similar articles [44,45]. The results from zeta potential analysis showed a concentration-dependent increase in mean zeta potential where at higher concentration of 1 mg/mL peptide the CT25 B surface charge changes to positive. This zeta potential increase could result in enhanced destabilization of CT25 B formulation due to a decrease in repulsion forces leading to the observed increase of PDI values. In addition to this, this surface charge increase corroborates with peptide adsorption in a concentration-dependent manner. Both peptides are characterized to have a positive net charge which could explain this increased surface charge. On the other hand, incubation of Lys(N_3_)-MAP and NPY on loaded drug NLC did not change the overall values when compared to the values obtain for CT25 B. Zeta potential confirmed peptide adsorption in all NLCs irrespective of the drug being associated or not.

The use of different incubation medium was also evaluated, and results demonstrated high differences between H_2_O dd and PBS incubation. In the physiological state with PBS after peptide incubation we observed a significant increase in particle size allied with increase in PDI values. This data suggest that in PBS, Lys(N_3_)-MAP adsorption led to NLC aggregation in suspension (Scheme 2—aggregation hypothesis). Several studies demonstrated that MAP tends to aggregate in monomers when close to lipidic cell membranes [28,46,47]; similar mechanism might be involve due to lipidic proprieties of the NLC formulation. The data resulted from zeta potential analysis showed some differences from PBS incubation when compared to H_2_O dd incubation. Indicating that this functionalized NPs have different physicochemical proprieties in physiologic conditions which will result in different behavior in a possible systemic administration. These results demonstrated the importance to test formulations in different physiologic conditions which is crucial to understand and predict the type of behavior our NPs delivery systems will have when administrated in vivo. Furthermore, with this method, zeta potential shows that NPY was successfully adsorbed into CT25 B and drug loaded CT25. The zeta potential values indicated the NPY surface adsorption led to more stable NLC formulations due to higher mean zeta potential value obtained (Scheme 2—disperse formulation). This initial analysis demonstrated promising results with successful adsorption of these three peptides onto NLC surface. Still, more experiments are needed to be conducted to better understand the types of interactions that are occurring. By analyzing the proprieties and structure of both peptides and NLC components we can propose two ways of adsorption that might be happening. First, the polyethylene oxide chains-exposed hydrogen groups can interact with amine groups present in the peptide sequences leading to the formation of hydrogen bonds. The second hypothesis is the interaction of NLC lipophilic part with peptides by the formation of hydrophobic bonds or through van der Waals attractive forces. Taking in consideration the isoelectric points, net charge, and average hydrophilicity of Lys(N_3_)-MAP and NPY proprieties we could predict that adsorption occur on the negatively charged NLC surface. Furthermore, several studies have demonstrated the use of tween 80 in the NLC formulation has an important role in adsorption of human plasma proteins in particular apolipoprotein E that can promote transport across blood brain barrier through low density receptor [48,49,50].

In the second part of the work, we evaluate the possible advantages from functionalization of the NLC surface with in silico evaluation of interaction between NPY Y1 receptor and MAP. The structural information of the results obtained from the complex docking in silico experience allows to clarify the mechanism of molecular recognition and a possible mode of interaction that could reflect the observed in vitro results in cells. Biochemical effects of an agonist and antagonist differ and produce different responses in the cells. Usually, agonists stabilize the receptor conformation in the active form whereas antagonists stabilize the receptor conformation in the inactive form and interfere with the binding of agonists [51]. The overall results demonstrated strong association between MAP and NPY Y1 receptor when compared to both UR-ML299 antagonist and NPY. Moreover, docking assays demonstrated that MAP interacts through N-terminal amino acid residues with deep insertion into NPYR1 inside cavity such as the UR-ML299 antagonist. More studies must be included to better understand the possible cellular outcome that can arise from this MAP interaction with the NPY 1 receptor and important to evaluate if MAP has the capability to interact to other NPY receptors. These studies can be crucial to determining the advantage of this adsorption in the NLC surface.

## 4. Materials and Methods

### 4.1. Materials

Tacrine hydrochloride (9-amino-1,2,3,4-tetrahydroacridine hydrochloride hydrate) (TAC), Tranylcypromine ((±)-trans-2-Phenylcyclopropylamine) (TCP), Rasagiline mesylate (N-Propargyl-1(R)-aminoindan methanesulfonate) (RAS), and buffer solution (PBS—pH 7.4) were acquired from Sigma-Merck (Merck Life Science S.L.U., Algés, Portugal). Diethylene glycol monoethyl ether (Transcutol HP^®^) and glyceryl dibehenate (Compritol^®,^ 888 ATO) were a kind gift from Gattefossé (Neuilly-Sur-Seine, France). Polysorbate 80 (Tween 80^®^) was purchased from J. Vaz Pereira S.A. (Sintra, Portugal). Neuropeptide Y was Sigma-Merck (Merck Life Science S.L.U., Algés, Portugal). Peptides MAP and Lys(N_3_)-MAP, TAC-MAP, and RAS-MAP were previously synthetized by our research group as described in [52,53]. Briefly, MAP was synthetized through Fmoc/tBu solid-phase peptide synthesis (SPPS) methodologies assisted with microwave (MW) energy, using a Liberty Microwave Peptide Synthesizer. Conjugation with TAC or RAS was achieved through “click chemistry” −a classical copper-catalyzed azide−alkyne cycloaddition (CuAAC) reaction.

### 4.2. Nanoparticle Preparation

Blank, RAS-loaded, TAC-loaded, and TCP-loaded NLC were already prepared in a previous work with modification of a hot high shear homogenization (HSH) method described at [53,54]. Around 300 mg of Compritol 888 ATO was melted at a temperature of 80 °C. RAS, TAC, and TCP were dissolved in the liquid lipid Transcutol HP (at theoretical concentrations of 25% *w*/*w*), and then added to the molten solid lipid. A hot aqueous phase consisting of 10 mL ultra-purified water with 3% of Tween 80 was added to the lipid phase under high-shear homogenization at 12,300 rpm for 10 min on a Silverson SL2 (UK), in a water bath to maintain 80 °C. Afterwards, the NLC dispersion was cooled in an ice bath with gentle stirring for 5 min. Each formulation was carried out in triplicates (*n* = 3). The final dispersion was sealed and stored at 4 °C until further use.

### 4.3. Peptide and Peptide Conjugate Adsorption Method

NPY, Lys(N_3_)-MAP, and TAC-MAP conjugate adsorbed onto NLC. The method was carried out with the incubation of 150 µL and 300 µL of NLC formulation (Blank and/or drug encapsulated) with different peptide solution: NPY (0.2 mg/mL) in ultra-purified water; Lys(N_3_)-MAP (0.1–1 mg/mL) in ultra-purified water and (0.5 mg/mL) in PBS pH 7.4; TAC-MAP (0.1–1 mg/mL) in ultra-purified water and (0.5 mg/mL) in PBS pH 7.4, at 37 °C, overnight with gentle horizontal shaking (300 rpm).

### 4.4. Nanoparticle Size and Surface Charge

The average particle size of NLC formulations before and after peptide adsorption was analyzed by photon correlation spectroscopy (PCS) using a Zetasizer Nano S (Malvern Instruments, UK). Measurements were made at a scattering angle of 173°, at 25 °C after appropriate dilution (1:5) in filtered purified water. All results obtained were expressed as average particle size and polydispersity index (PDI). Furthermore, zeta potential determination was performed by anemometry using a Zetasizer Nano Z (Malvern Instruments, Malvern, UK). For that purpose, samples were placed in a specific cuvette where a potential of 150 mV was established after appropriate dilution (1:5) of the samples in filtered purified water. For all the measurements, three replicate samples were determined.

### 4.5. Statistical Analysis

Statistical analysis of the experimental data was performed using a two-way analysis of variance (two-way ANOVA) and differences between groups were tested by a two-way ANOVA with GraphPad Prism version 8.0 (GraphPad Software, San Diego, CA, USA). Data were expressed as mean SD or 95% confidence interval. A *p* < 0.05 value was considered significant. All data are shown as mean SD.

### 4.6. Receptor Molecular Docking Assays

#### 4.6.1. Model Preparation for Human Neuropeptide Y Y1 Receptor

A model for the human neuropeptide Y receptor type 1 (NPY1R) was prepared, starting from the 5ZBQ [55] crystallographic structure available on the Protein Data Bank [56] with a resolution of 2.70 Å. This structure represents NPY1R complexed with T4 Lysozyme and with the selective antagonist ligand UR-MK299 [55]. In preparing the NPY1R model, the T4 lysozyme was removed, while UR-MK299 was extracted to be used as reference ligand for docking validation. Missing amino acid residue position (241 to 256) were modelled upon alignment with the Alphafold Model for this receptor [57].

CHARMM-GUI Membrane Builder [58] was used to position NPY1R into a 100 × 100 Å membrane bilayer, comprised of 145 cholesterol molecules, 149 1-palmitoyl-2-oleoyl-sn-glycero-3-phosphoethanolamine (POPE), and 135 palmitoylsphingomyelin (PSM) molecules, in agreement with the GPI-Anchored CD59 in human plasma membrane described by Lee et al. [59].

#### 4.6.2. Preparation of MAP and NPY

Structures for MAP and NPY were prepared with AMBER21 software [60] using the xleap module and GaussView 5.0. Structures were placed in TIP3P periodic water boxes with a minimum distance of 12 Å of waters to the box edges. Periodic boundaries were applied, and the long-range electrostatic interactions were calculated using the particle mesh Ewald summation method. The cut-off value for the short-range electrostatic and Lennard–Jones interactions were set at 10.0 Å. The hydrogen bonds were constrained using the SHAKE algorithm. A time step of 2 fs was used.

Four minimization steps were performed to remove clashes and applied in the following order: 1. water molecule (2500 steps); 2. hydrogens atoms (2500 steps); 3. side chains of all the amino acid residues (2500 steps); and 4. full systems (10,000 steps). After minimization, a molecular dynamics equilibration procedure was performed for 50 ps, in which the systems were gradually heated to 310 K using a Langevin thermostat at constant volume (NVT ensemble). Finally, the production phase was run for a total of 1000 ps in an NPT ensemble at a pressure of 1 bar and a temperature of 310 K. This protocol is described in more detail in the literature [61,62,63]. Stabilized conformations of MAP and NPY in water were taken from the equilibrated stage of both MD simulations and were used as starting point for flexible-peptide docking calculation.

#### 4.6.3. Docking

UR-MK299 antagonist was used as a reference to evaluate the accuracy of the docking protocol and to optimize it. The conformation of this ligand was randomized, and the ligand was redocked against the NPY1R-membrane model structure using the GOLD software [64] with the PLP scoring function [35]. The binding pocket was defined as centered on the initial cavity defined by UR-MK299, plus a radius of 30 Å, encompassing the NPY1R amino acid residues in the outer membrane leaflet. The optimized protocol was later extended to enable the docking of MAP and NPY models.

## 5. Conclusions

In conclusion, through this preliminary experiment we were able to conduct an initial analysis of the influence of different parameters and peptides on the adsorption in lipid nano systems surface. The results demonstrated great potential of nanoparticle surface functionalization with different peptides, including a modified CPP MAP, NPY, and TAC-MAP conjugate. In previous results conducted by our group, we have demonstrated a possible advantage with the use of MAP and conjugate TAC-MAP alone and loaded in NLC in vitro with neuroblastoma cell line SH-S5Y5, the results demonstrated a cellular response depending on the concentration administered to the cells [52,53,65]. In addition, the results presented in this paper are important to complement previous work conducted and show the multiple functionalizations that can be applied when designing and optimizing a nanodelivery system. For instance, the use of CPP functionalized NLC loaded with drug have been demonstrated that might promote increased cell penetration against a variety of diseases dependent on the drug encapsulated [66,67,68,69,70]. Moreover, the use of drug-loaded NLC functionalized with NPY might open the possibility in future studies to understand the impact of therapeutic delivery in neuronal disorders on both in vitro and in vivo models [71]. Moreover, in the second part of the paper, the in silico analysis demonstrated strong interaction of MAP against NPY receptor. In future studies we want to determine peptide adsorption stability on the NLC in different conditions, quantify the amount of peptide in NLC surface to study its possible use with in vitro and/or in vivo neurodegenerative diseases models.

## Figures and Tables

**Table 1:** Resume data analysis from all the adsorption experiments conducted in this article.

NLC	Incubation Conditions *	PS (nm) *	PDI *	ZP (mV) *
CT25 B	H_2_O dd	140.1	0.259	−7.88
CT25 B (300 µL)	H_2_O dd	146.5	0.265	−8.53
CT25 B	Lys(N_3_)-MAP 0.1 mg/mL in H_2_O dd	160.5	0.281	−1.89
CT25 B	Lys(N_3_)-MAP 0.5 mg/mL in H_2_O dd	169.1	0.285	−0.05
CT25 B	Lys(N_3_)-MAP 0.8 mg/mL in H_2_O dd	171.8	0.287	0.85
CT25 B	Lys(N_3_)-MAP 1 mg/mL in H_2_O dd	173.7	0.297	1.27
CT25 TAC	H_2_O dd	159.5	0.258	−9.73
CT25 RAS	H_2_O dd	104.8	0.210	−9.39
CT25 TCP	H_2_O dd	144.5	0.254	−9
CT25 TAC	Lys(N_3_)-MAP 0.8 mg/mL in H_2_O dd	200.5	0.305	1.28
CT25 RAS	Lys(N_3_)-MAP 0.8 mg/mL in H_2_O dd	112.6	0.251	1.23
CT25 TCP	Lys(N_3_)-MAP 0.8 mg/mL in H_2_O dd	177.3	0.287	1.85
CT25 B	TAC-MAP 0.1 mg/mL in H_2_O dd	158.3	0.288	−1.65
CT25 B	TAC-MAP 0.5 mg/mL in H_2_O dd	164.6	0.270	0.16
CT25 B	TAC-MAP 0.8 mg/mL in H_2_O dd	167.6	0.272	0.3
CT25 B	TAC-MAP 1 mg/mL in H_2_O dd	165.9	0.287	0.55
CT25 B (300 µL)	TAC-MAP 0.5 mg/mL in H_2_O dd	164.9	0.296	−0.54
CT25 B	PBS (pH 7.4)	141.2	0.264	−7.82
CT25 B	Lys(N_3_)-MAP 0.5 mg/mL in PBS PBS (pH 7.4)	267.8	0.887	2.83
CT25 B	TAC-MAP 0.5 mg/mL in PBS PBS (pH 7.4)	217.6	0.350	−2.16
CT25 B	NPY 0.2 mg/mL in H_2_O dd	143.1	0.269	−23.27
CT25 TAC	NPY 0.2 mg/mL in H_2_O dd	160.4	0.261	−25.20
CT25 RAS	NPY 0.2 mg/mL in H_2_O dd	105.3	0.222	−21.73

* H_2_O dd—double distilled ultra-purified water; TAC—Tacrine; RAS—Rasagiline; TCP—Tranylcypromine; B—blank; PBS—phosphate buffered saline; PS—particle size; PDI—polydispersity index; ZP—zeta potential.

**Table 2:** Docking scores of UR-MK299, MAP and NPY on NPY1R.

	UR-MK299	MAP	NPY
GOLD/PLP Score	107.70	101.29	95.12

## Data Availability

Not applicable.

## Sample Availability

Samples of the compounds are not available from the authors.
